# Robot-Assisted Minimally Invasive Breast Surgery: Recent Evidence with Comparative Clinical Outcomes

**DOI:** 10.3390/jcm11071827

**Published:** 2022-03-25

**Authors:** Kuo Chen, Jin Zhang, Narasimha M. Beeraka, Mikhail Y. Sinelnikov, Xinliang Zhang, Yu Cao, Pengwei Lu

**Affiliations:** 1 Department of Breast Surgery, The First Affiliated Hospital of Zhengzhou University, 1 Jianshedong Street, Zhengzhou 450052, China; chenkchenk@foxmail.com; 2 Department of Pharmacology and Toxicology, University of Mississippi Medical Center, Jackson, MS 39216, USA; alexander600229@gmail.com; 3 Department of Human Anatomy, I.M. Sechenov First Moscow State Medical University, Ministry of Health, Russian Federation, 8/2 Trubetskaya Street, 119991 Moscow, Russia; mikhail.y.sinelnikov@gmail.com (M.Y.S.); zhangxinliang980620@gmail.com (X.Z.); goodmancaoyu@gmail.com (Y.C.); 4 Center of Excellence in Molecular Biology and Regenerative Medicine (CEMR), Department of Biochemistry, JSS Academy of Higher Education and Research (JSS AHER), JSS Medical College, Mysuru 570015, India

**Keywords:** robot-assisted surgery, open surgery, postsurgical complications, ergonomics

## Abstract

In recent times, robot-assisted surgery has been prominently gaining pace to minimize overall postsurgical complications with minimal traumatization, due to technical advancements in telerobotics and ergonomics. The aim of this review is to explore the efficiency of robot-assisted systems for executing breast surgeries, including microsurgeries, direct-to-implant breast reconstruction, deep inferior epigastric perforators-based surgery, latissimus dorsi breast reconstruction, and nipple-sparing mastectomy. Robot-assisted surgery systems are efficient due to 3D-based visualization, dexterity, and range of motion while executing breast surgery. The review describes the comparative efficiency of robot-assisted surgery in relation to conventional or open surgery, in terms of clinical outcomes, morbidity rates, and overall postsurgical complication rates. Potential cost-effective barriers and technical skills were also delineated as the major limitations associated with these systems in the clinical sector. Furthermore, instrument articulation of robot-assisted surgical systems (for example, da Vinci systems) can enable high accuracy and precision surgery due to its promising ability to mitigate tremors at the time of surgery, and shortened learning curve, making it more beneficial than other open surgery procedures.

## 1. Introduction

According to GLOBOCAN 2018, breast cancer is one of the most common malignant tumors occurring in women, and considered to be the leading cause of mortality compared to all other cancers in gynecology and obstetrics fields. Global five-year incidence of breast cancer is more than 43 million cases. The incidence rate in northern Europe is 25.9% per 100 thousand women, whereas the rate is 90.1%, and 94.2% in central Asia and Australia, respectively [1,2,3,4]. The progression of breast cancer from stage 0 to IV is aggressive, due to the higher invasion and metastasis ability of the cancer cells to invade vital organs, such as the brain and liver, through blood or lymphatic circulation, seriously threatening the patient’s overall quality of life.

At present, surgery is one of the main approaches for breast cancer treatment [5,6,7,8]. However, the complications of conventional surgery include lymphedema, fat necrosis, wound infection, range-of-motion restriction, and arm paraesthesia [7,9,10,11,12,13,14]. Robot-assisted systems for oncologic surgery are approved by the US Food and Drug Administration (FDA) for specific abdominal surgical interventions. Robot-assisted surgery [4] is a recent trend that constitutes a leap forward in minimally invasive breast surgery. Furthermore, it is significantly used in other surgical interventions, including breast, thyroid, urological, colorectal, prostate, pediatric, gastrointestinal, and gynecological procedures [15,16,17,18,19,20,21,22,23,24,25]. For instance, nipple-sparing mastectomy is improved by using robots [26]. Ind et al. showed a robot-assisted laparoscopic surgery that has more favorable clinical outcomes than conventional surgery for endometrial carcinoma [27]. Hazey et al. described the laparoscopy application of robot-assisted technology in general procedures [28]. Thus, robot-assisted surgery seems to be a promising way to attain patient safety, high efficacy and precision surgery. In this review, we substantially discussed the significant implications of robot-assisted, minimally invasive, breast surgery and comparative clinical outcomes with robot-assisted breast surgery in breast cancer patients in relation to other cancers receiving robot-assisted surgery.

### Plan of Work

We searched public databases, such as Pubmed, Medline, the National Library of Medicine, and Google Scholar for published reports pertinent to successful clinical outcomes of robot-assisted surgery. We searched these public database platforms using keywords, including robot-assisted surgery, da Vinci robot surgery system, breast surgery, latissimus dorsi breast reconstruction, and nipple-sparing mastectomy, complication rates during robot-assisted surgery, microsurgeries, direct-to-implant breast reconstruction, and deep inferior epigastric perforators-based surgery. The information was accessed on dates between 8 July 2021 to 12 December 2021.

## 2. Robot-Assisted Surgery

**Figure 1:**
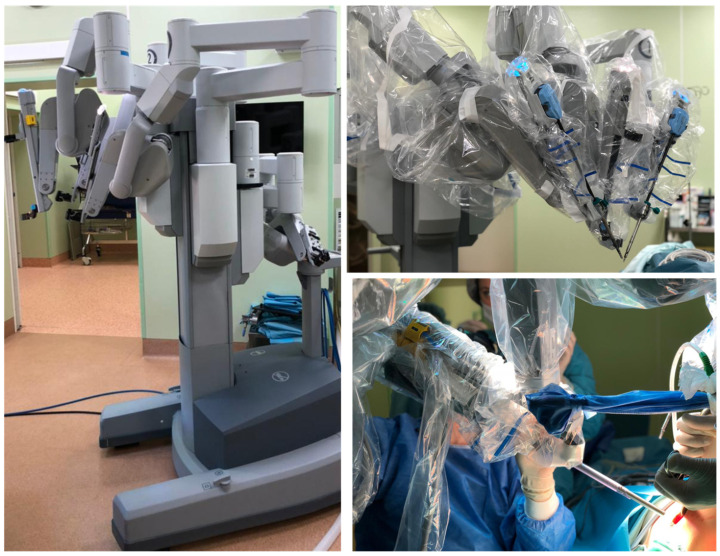
Schematic depiction of robot-assisted surgery (example: da Vinci robot-assisted surgical system) equipped with easily maneuverable, flexible, and stable mechanical arms. The implications of the robot-assisted surgery system can assist the surgeon to perform surgery typically with substantial precision and can generate patient-satisfied aesthetic requirements according to the design of incision. Model: the latest da Vinci’s robot-assisted surgery model is “the Si”, released in 2009. It is composed of a master console, a mobile platform, and an operational cart with four arms; Company: Intuitive Surgical Inc., Sunnyvale, CA, USA; Date of image acquisition from the I.M. Sechenov First Moscow State Medical University of the Ministry of Health of the Russian Federation (Sechenov University), 8/2 Trubetskaya Street, Moscow, 119991, Russia: 29 September 2019.

The concept of using robots for remote operations was first developed by the US Army and National Aeronautics and Space Administration (NASA) [29]. Robot-assisted surgery has been able to improve surgical technique limited by the human body through several advantages, such as up to seven degrees of freedom, tremor elimination, 3D magnified vision, ergonomic positioning, and improved resolution. The free and more precise movements have led to rapid applications of robot-assisted surgery in various departments. The first application of robot-assisted surgery was for obtaining a stereotactic brain biopsy in 1985 with the Programmable Universal Machine for Assembly 200 (PUMA) [30]. After several years of development, during 1991, Davies et al. used PUMA 500 to remove prostatic tissue through transurethral resection of the prostate [31]. Nowadays, the da Vinci surgical robot has replaced PUMA and other earlier robots as the primary choice for surgeons all over the world, because it can provide three-dimensional, high-definition, and microscopic views. The flexible and versatile robot arms in any robot-assisted surgery can operate precisely within a minimal incision, then reach into a narrow surgical site (example: axilla, pelvis) and accomplish most surgical procedures safely and effectively, with minimal invasion, to reduce the incidence of complications and generate patient-specific aesthetic effects [18,32,33,34] (Figure 1). Due to the development of network communication technology, rapidly emerging robot-assisted surgery can be combined with fifth-generation wireless networks (5G) to develop safe and effective implementation of remote surgery and telementoring, breaking through the limitation of time and space, greatly improving efficiency and preventing patients having to make long-distance trips [35,36].

### 2.1. Robot-Assisted Nipple-Sparing Mastectomy

Traditional radical mastectomy not only removes breast and axillary lymph node tissue, but also removes chest wall muscle tissue and covering skin. After radical mastectomy, the patients are healed by a skin graft or secondary intention, leaving deformed and sunken chest wall deformities. Half a century ago, Madden et al. published reports pertinent to the development of modified radical mastectomy, and concluded that preserved chest wall muscles and adequate skin can usually achieve primary closure [37]. Modified radical mastectomy reduces the incidence of infections and other complications by preserving the chest wall muscles, but most of the breast skin and nipple areola complex are removed. The application of skin-sparing and nipple-saving mastectomy can greatly improve aesthetic appearance, while still maintaining oncology effects [38]. A large number of studies have been conducted to prove the safety of skin-sparing mastectomy, showing that the local recurrence rate of skin-sparing mastectomy is equivalent to that of non-skin-sparing mastectomy [39,40,41]. A more natural and beautiful breast shape can be reconstructed with good aesthetic effect induced by saving the skin envelope and breast pocket. The nipple-sparing mastectomy is a well-advanced version of skin-sparing mastectomy by preserving the nipple areolar complex, but this kind of mastectomy removes breast tissue and nipple areolar ducts. A frozen section of subareolar tissue is usually sent to confirm negative margins. Atypia or positive surgical margins lead to conversion to a skin-sparing mastectomy which ranges from 2.5–12% [42,43]. Even more gratifying are the survival and local recurrence rates of nipple-sparing mastectomies, which are as good as those of either skin-sparing or modified radical mastectomies [44,45]. Moreover, the aesthetic effects brought by nipple-sparing mastectomies have improved patient satisfaction [46,47].

Robot-assisted nipple-sparing mastectomy was first proposed by Toesca et al. in 2015, and this system can perform nipple-sparing mastectomy at a single axillary scar, with immediate reconstruction using robotic implants [48]. One year later, Sarfati et al. also demonstrated technical feasibility with a small incision cadaver study using robot-assisted surgery [49]. Toesca et al. reported a 29-case series of robot-assisted nipple-sparing mastectomy, which can be executed in approximately 3 hours; the procedure resulted in a low conversion to open rate accomplished with acceptable feasibility, reproducibility, and safety without major complications, like infections [50]. Another significant aspect in this kind of surgery is that intermittent discharge of carbon dioxide and placement of wet and cold gauze on the mastectomy flap can prevent high carbon damage [51]. In order to ensure the safety and effectiveness of robot-assisted mastectomy, significant personnel training is required in order to perform robot-assisted surgery. This kind of minimally invasive approach, with improved aesthetics, for nipple-sparing mastectomy can enhance the overall quality of a patient’s life. Both the oncologic and aesthetic requirements after this surgical intervention are described in Table 1.

### 2.2. Robot-Assisted Latissimus Dorsi Breast Reconstruction

The latissimus dorsi flap was first described by IginioTansini in the 1900s [52]. The incision in this procedure usually ranges from 15 to 45 cm. In order to improve the incision, endoscopy could be applied [53,54]. In 2011, Selber et al. primarily described a robot-assisted harvest of a latissimus dorsi muscle for breast reconstruction through a cadaveric feasibility study [55]. Furthermore, Selber et al. reported a follow up of a total of a seven-case series that underwent breast reconstruction through robot-assisted harvest of latissimus dorsi muscles [56]. As per this study, there were no significant complications, while the robot-assisted harvest time decreased from 2 hours to 1 hour. The latissimus dorsi muscle is the largest muscle in the body. The classic open flap harvest technique of this muscle results in a long posterolateral thoracic vertical oblique incision that can leave an unappealing scar. The minimally invasive robot-assisted approach has the potential to reduce scar length and to overcome technical limitations of endoscopic techniques. Fouarge et al. confirmed that robot-assisted latissimus dorsi muscle flap harvest is a safe, reproducible, and effective tool, that offers precise dissection control and leaves a minimal thoracic scar [57].

### 2.3. Robot-Assisted Surgery—Deep Inferior Epigastric Perforator Flap

The deep inferior epigastric perforator flap is a standard surgical intervention in breast reconstruction. Autologous-based breast reconstruction always produces the most satisfactory results in patients’ overall quality of life [58,59,60,61,62,63,64,65]. However, after the execution of deep inferior epigastric perforator-based surgery, complications like muscle bulging may damage segmental rectus nerves; therefore, nerve preservation is important to reduce muscle bulge [66]. By means of robot-assisted surgery, the fascial incision can be significantly limited, which may reduce the incidence of muscle bulging and nerve damage [67,68]. Manrique et al. described a study about the practice of robot-assisted deep inferior epigastric perforator on a cadaveric model, which may help surgeons to improve skills and increase the success rate [69]. Thus, several studies concluded that deep inferior epigastric perforator surgery is a significant standard method to promote breast reconstruction, due to its ability to confer the natural shape, and maintain permanence of static and dynamic symmetry. Efficacy of deep inferior epigastric perforator flap-reconstructed breast surgery can be ascertained with neurotization and non-neurotization, as deep inferior epigastric perforator flap surgery can increase the spontaneous recovery of sensory innervations across the reconstructed breast with minimal postoperative complications. For instance, breast sensibility is provided through the 3^rd^ to 6^th^ intercostal nerves as well as the supraclavicular branches of the cervical plexus. Radical mastectomy could sever these intercostal nerves, but deep inferior epigastric perforator flap through robot-assisted surgery could harvest these sensory branches and anchor lymph node vascularization across the skin paddle in the reconstructed breast. The ability to neurotize flaps may enhance spontaneous sensory recovery after a 6 to 12 months postoperative period. Therefore, the deep inferior epigastric perforator flap-mediated reinnervation can be achieved through robot-assisted surgery to foster neo-breast integration in body image and consequently enhance self-esteem and patient quality of life.

### 2.4. Robot-Assisted Direct to Implant Breast Reconstruction

According to a study by Parcells et al., a robot-assisted surgical approach for ‘direct to implant’ reconstruction with an acellular dermal matrix scaffold is feasible and addresses limitations with open approaches and ergonomics. The authors performed a cadaveric exploration to demonstrate proof of concept and feasibility for a robot-assisted direct to implant reconstruction following a robot-assisted nipple-sparing mastectomy. Tremor stabilization, direct visualization, endo-wristed robotic instrumentation, and exposure were noted as key benefits over existing open ‘direct to implant’ reconstruction techniques. Additionally, the ability to have more remote access to entry at the perimeter of the breast eliminated incisional tension, which can normally jeopardize reconstructive results [70] (Table 2).

## 3. Robot-Assisted Surgery in Breast Cancer and Breast Reconstruction: Clinical Outcomes

The microsurgical competency of a robot system can effectively harvest intermammary vessels during breast reconstruction [71]. This surgical intervention is similar to the technique used in cardiac surgery and then followed by a traditional free flap strategy. During this breast surgery, removal of intercostal cartilage was avoided and it allowed a mean pedicle length of 6.7 cm, which was covered by the intercostal muscle of the 2nd intercostal space. For instance, the mean surgical time for harvesting the intermammary vessels was 113 minutes but the average hospital stay was seven days accompanied by a 3-day stay in the intensive care unit during the postoperative period. However, the complications rate was observed in 6 out of 22 patients having to be returned to the surgery theater in order to remove hematoma, a critical postoperative complication. The traditional open technique for harvesting the latissimus dorsi flap in breast reconstructive surgery can result in a lengthy and unsightly scar. Therefore, a laparoscopic technique was developed. The da Vinci robot-assisted system can produce enhanced 3-dimensional visualization. Furthermore, this kind of robot-assisted surgery is operated by surgical dexterity with a wider wrist range of motion than the laparoscopy method during breast reconstruction. As we discussed, Selber et al. employed a cadaveric model in order to harvest latissimus dorsi muscle flaps and the average incisional length can be 5 cm for three ports and the robot docking time approximately 23 minutes with average harvest time of 68 minutes. Later, the reproducibility and feasibility were examined by executing this robot-assisted surgery in a clinical series with the successful harvest of seven latissimus dorsi muscle flaps, five of which were used for breast reconstruction. In this study, only one clinical case of complication with transient radial nerve palsy was implicated, secondary to malposition [72]. Another study by Clemens reported reproducibility with robot-assisted latissimus dorsi harvest in two-stage delayed immediate breast surgery, once radiotherapy has been finished. However, a longer harvesting time was observed with this robot-assisted latissimus dorsi harvest than the ‘traditional open technique’, but the average hospital stay was less with robot-assisted surgery than with the ‘traditional open technique’. However, large epidemiological statistical data are yet required to conclude the efficiency of robot-assisted latissimus dorsi harvest in breast surgery by studying a large patient cohort.

## 4. Robot-Assisted Microsurgery

The unique features of the robot-assisted system, such as complete tremor elimination, 5:1 motion scaling, 10× magnification, and high-dimensional optics are considered as crucial for the successful implementation of microsurgery and supermicrosurgery. For example, robot-assisted microvascular anastomosed surgeries, like robot-assisted lymphovenous bypasses for lymphedema surgery, are becoming more popular day by day [73,74]. The robot setup in this procedure allows the microsurgeon to operate at the console in an ergonomic position. In addition to microvascular surgery, surgical robots can also increase the success rate of repairing peripheral nerves [75,76,77]. Using the robot-assisted technique in brachial plexus surgery can avoid a long incision and subsequent dissection. The routine application of minimally invasive robot-assisted surgery could eventually enable earlier diagnosis and treatment for brachial plexus injuries [76]. The usage of lymphovenous bypass was first described by O’Brien et al. in 1977 in dogs [78]. However, decades were taken in developing supermicrosurgical techniques with sophisticated instrument improvements to enable lymphovenous bypasses to come into the armamentarium of microsurgeons. Koshima et al. described the supermicrosurgery procedure, which performs anastomoses on calibers of 0.3–0.6 mm, enabling surgical treatment of lymphedema with lymphovenous bypasses [79]. Lymphovenous bypasses are typically performed in an end-to-end fashion using 11-0 or 12-0 nylon sutures with a 50-µm needle [80]. These anastomoses challenge, and can even surpass, the limits of human precision. Even a subtle tremor is exploited by the extreme magnification. The absence of tremor in the robot during surgical intervention is especially beneficial in supermicrosurgery. Indocyanine green angiography is often used when mapping and planning lymphovenous anastomoses and to prove patency. The robot-assisted platform allows facile transitioning between near infrared and normal bright field vision, which is another significant advantage. Another study described the application of the da Vinci robot-assisted surgery system in order to perform multiple lymphovenous anastomoses successfully and concluded that there are significant benefits of this robot system for supermicrosurgery. Recently, the world’s first dedicated robot-assisted platform for (super) microsurgery, MicroSure (MicroSure, Eindhoven, The Netherlands) has been developed [81]. This is designed to aid stabilizing movements during microsurgery by filtering tremors and scaling down motions. This robot-assisted surgery system is easily maneuverable, and equipped with arms holding genuine microsurgical instruments that are easily placed into the holders, and are compatible with conventional surgical microscopes. Preclinical tests of this robot-assisted system have confirmed the safety and feasibility of this robot in performing microsurgical anastomosis [34,82]. Several types of breast surgeries vividly executed using these robot-assisted surgical systems are described in Table 3.

Robot-assisted surgery was also implicated in breast cancer patients suffering from axillary lymph node metastasis due to internal mammary lymph nodes [93]. Parasternal radiotherapy can cause defects in functional aspects of the majority of vital organs which can reduce overall quality of life in these patients [94,95,96,97]. Hence, robot-assisted surgery can be implicated to remove the internal mammary lymph nodes in breast cancer patients. Most importantly, three-dimensional imaging facilities in this robot-assisted system can provide clearer vision than open surgery or other traditional surgical methods. It is composed of an operating instrument combined with a special internal joint with 7° of freedom; this kind of special operating system can promote the ability of surgeons to execute surgical procedures with utmost accuracy and reliability [93].

Endoscopic-assisted surgery can facilitate breast cancer patients with good aesthetic effects and long-term therapeutic safety [71,98,99]. At present, robot-assisted surgery (for example, da Vinci) has been significantly recommended for metastatic cancers using surgical interventions for urinary surgery, gynecology, and thyroid surgery [100,101,102]. Robot-assisted breast reconstruction may produce good clinical outcomes and enhance the overall quality of life in breast cancer patients by minimizing overall surgical complications [103,104,105].

Patient satisfaction rates are reported to be higher in these robot-assisted surgery procedures. For instance, the da Vinci robot-assisted surgery is one of the significant robot-assisted surgery systems that could be used in male mastectomy and internal mammary lymph node biopsy [106]. A recent study performed by us reported the efficacy of robot-assisted surgery to ascertain the minimizing efficacy of postoperative complications [4]. In the case of da Vinci robot-assisted surgery, a significant 3D surgical view can enable the surgery process, due to the presence of flexible and stable mechanical arms to operate, typically with higher precision and minimal incision. It is easier to identify the blood vessels as well as the lymph nodes with higher precision, as the robotic arms can satisfy aesthetic requirements as per the design of the incision. Thus, the overall surgery time can be mitigated at the time of removing lymph nodes as it is possible to apply grasping forceps and ultrasound knives in order to excise the axillary lymph nodes in the chosen breast cancer patients [17]. In addition, damage to the nerves, and surrounding blood vessels is substantially minimalized, as the whole robot-assisted surgical system can be significantly used, even with minimal surgical incision. Therefore, the patient can recover with very minimal complications, like wound infection and fat necrosis, and with minimal lymphedema of upper limbs. In our recent study, we have observed incisions as aesthetically pleasing, typically due to less traumatization and lack of hematoma-related complications [4].

## 5. Comparative Efficacy in Clinical Outcomes of Robot-Assisted Surgery for Breast Cancer over Other Cancers

Robot surgery can bestow significant advantages when compared to conventional laparoscopy [107]; and patients attained significantly shorter stays and minimal blood loss after this surgery. In addition, minimal conversion of laparotomy rate and adequacy of surgical staging are predominantly in favor of the robot-assisted surgery [108,109,110,111,112,113]. It has been observed that superior ergonomics and mitigated tremors can be enhanced with robot-assisted surgery, and this kind of surgical intervention typically minimized the learning curve [4,109,110,111,112,113,114,115]. In the case of obese patients, it is apparently challenging for open or conventional type surgery, as the patients may experience higher postoperative complications; hence, da Vinci robot-assisted surgery can ensure that these obese patients experience minimal postoperative complications [116]. Robot-assisted surgery is crucial for elderly patients suffering from endometrial cancer who often cannot cope with a steep Trendelenburg position; hence, significantly higher pressure is required for abdominal insufflations because of their comorbidities. These demands can be mitigated with robot-assisted surgery [115,117,118,119]. In addition, conventional surgery can cause mental fatigue and musculoskeletal ailments among surgeons who are regularly executing surgical interventions [120,121,122]. Occupational symptoms are predominantly higher in surgeons who are recurrently performing minimally invasive surgery [123,124,125,126,127]. According to the guidelines of the FDA, robot-assisted surgery (for example, da Vinci surgery systems) was approved for both abdominal surgery and radical prostatectomy pelvic surgery [15] due to its substantial range of applications in urological and gynecological surgical interventions [16], mainly for prostatectomy, hysterectomy, and cholecystectomy. Significant reductions in intraoperative blood loss, total hospital stay, and risk of positive resection margins were observed in robot-assisted breast surgery when compared to other conventional procedures for other cancers, as well as robot-assisted surgical procedures for prostatectomy, hysterectomy, and cholecystectomy [4,128,129]. In addition, robot-assisted surgery application is widely elaborated for surgical intervention during head and neck cancers, and thyroidectomies [17,130,131,132].

However, a lot more retrospective studies are required to ascertain the comparative benefits of robot-assisted breast surgery with other minimally invasive surgeries, such as abdominal radical hysterectomy. For instance, a study by Pedro T Ramirez et al. (2018) reported the efficacy of robot-assisted surgery in women with early-stage cervical cancer, such as IA2, or IB1 cervical cancer, by segregating patients to undergo minimally invasive surgery or open surgery [133]. In this study, a total of 319 patients were categorized into minimally invasive surgery, whereas 312 patients were assigned into open surgery. The two groups exhibited nearly similar histologic subtypes, tumor size, and lymphovascular invasion. In addition, other properties, such as parametrial and lymph node involvement, are also similar in the two groups. Another study by PedjaCuk et al. (2021) compared the efficacy of robot-assisted colorectal surgery and laparoscopic colorectal surgery in terms of conversion rates, intraoperative blood loss, and morbidity and retrieval of bowel function [134]. A minimal conversion rate was observed in groups with ‘robot-assisted colorectal surgery’ due to the predominance of resection performed at the colon region. However, the conversion rates observed in patients undergoing ‘laparoscopic colorectal surgery’, in combination with neoadjuvant radiotherapy, were higher but non-significant. This kind of higher conversion rate is predominantly due to radiotherapy across the pelvic floor and the influence of factors such as fibrosis, tissue necrosis, and inflammation [135]. Enhanced robot-assisted colorectal surgery by the daVinci Si^®^ to the Xi^®^ model is one of the significant surgical interventions and this kind of process reported mitigation in the overall time of operation. Technical advances in robot-assisted surgery models can enable surgeons to perform colorectal surgery with more freedom due to its stable and high-precision camera and due to the presence of freely moving robotic arm joints to attain better hemostasis [136]. Furthermore, it has been observed that postoperative C-reactive protein levels are minimal during robot-assisted colorectal surgery conditions and easily predict inflammatory stress generated by surgery [137]. A recent observational study in Danish patients, who had undergone laparoscopic colorectal surgery (8104 cases) and robot-assisted colorectal surgery (511 cases) delineated higher risk of acquiring microradical resection in colon cancers with laparoscopic colorectal surgery. Yet, this was non-significant for the rectal cancer patients who had undergone surgical resection using robot-assisted colorectal surgery [138]. Thus, with technical advancements, telerobotics, and ergonomics, robot-assisted colorectal surgery could be more advantageous than laparoscopic colorectal surgery to enhance overall long-term survival with minimal traumatization [134]. The advantages and disadvantages of robot-assisted surgery compared to conventional surgery is given in the following Table 4.

## 6. Potential Barriers of Robot-Assisted Surgery in Clinical Application

The success of these robot-assisted surgery procedures is dependent on the operator and typically require long training periods for personnel to acquire specialized skills and training, which, in turn, increases labor costs. This could be a potential barrier for robot-assisted surgery interventions. Several studies also reported rapid boom in the usage of robot systems, although there is limited clinical rationale or theoretical benefit. Yet, a large amount of patient-based epidemiological data to support the usage of robot-assisted surgery is required in the clinical sector; this kind of population-based study is beneficial to characterize contemporary trends in the adoption of surgical robot-assisted systems for a wide range of surgical interventions [139,140,141,142,143].

The maintenance and repair of surgical robots requires a professional team of engineers. During the epidemic, many countries, including China, were without resident professional engineers. This made it difficult to repair surgical robots on time due to strict national isolation policies and visa restrictions, which could result in the deployment of surgical robots decreasing the success rate of selective surgical procedures [143]. At present, there are many companies supplying surgical robots with several brand names in the market. For instance, the latest da Vinci’s robot-assisted surgery model by Intuitive Surgical Inc., Sunnyvale, CA, USA i.e., “the Si” was released in 2009 and “the Xi”, was released in 2014. Both the models composed of a master console, a mobile platform, and an operational cart with four arms. The standardization of the available robots for the execution of effective breast surgery with utmost accuracy is yet to be deciphered through several studies. Lack of standardization and frequent software updates in the usage of these surgical robots can cause surgeons to take a longer time for recurrent learning, which will increase pressure on doctors and hospitals. The combination of surgical robots and technology of the 5G wireless network is another significant aspect to eliminate geographical restrictions that subsequently apply to deliver the services for patients in remote areas [143]. However, the risks of 5G infrastructures may itself add further risks for the remote application of surgical robots [143].

There are certain limitations for the usage of surgical robots during surgery; for instance, the robot may experience a mechanical failure or program failure during the operation. In this regard, traditional scalpels are more reliable than robots. Although the risk is very rare, there are times when a robotic machine can malfunction and cause more serious problems during surgery. In these rare instances, doctors have to quickly perform another type of surgical intervention to minimize or correct the damage. Possible burns, cuts or tears to the surrounding organs are other risks of the robot hand getting too close to another organ during surgery, causing damage.

## 7. Conclusions

The prospect of surgical robots is very bright, but technical difficulties associated with the development of robot-assisted systems require substantial observational studies in order to explore their wide range of applications on large population-based cohorts. Robot-assisted surgery can enable effective breast surgery and reconstruction accompanied by minimal hospital stay and complication rates that subsequently enhance overall quality of life. 

## Figures and Tables

**Table 1:** Robot-assisted nipple-sparing mastectomy: Clinical Outcomes in terms of complication rate and local recurrence.

Surgical Reports of Robot-Assisted Nipple-Sparing Mastectomy	Sample Size	Complication Rate	Local Recurrence	Reference
Medina-Franco H et al.	173 patients were included in this study	Not available	4.5% among all the patients	[38]
Agrawal A et al.	81 patients were included in this study	Not available	2% among all the patients	[39]
Kroll SS et al.	114 patients were considered in this study	Not available	7%	[40]
Gerber B et al.	60 patients were considered in this study	Not available	11.7%	[42]
Orzalesi L et al.	913 patients were considered in this study	4.4%	2.9%	[43]
Wijayanayagam A et al.	43 patients were included in this study	36%	Not available	[46]
Garwood ER et al.	106 patients were included in this study	11.8%	0.6%	[47]
Toesca A et al.	24 patients were included in this study	Not available	Not available	[48]

**Table 2:** Various studies pertinent to flap harvest, surgical operation length (in terms of time of operation), and overall hospital stay during robot-assisted breast reconstruction.

Surgery Reports of Robot-Assisted Breast Reconstruction	Sample Size (Number of Patients)	Flap Harvest (Mean) (Minutes)	Operation Length (Mean) (Minutes)	Hospital Stay (Mean) (Days)	Reference
**Latissimus dorsi breast reconstruction**					
Pomel C et al.	13	116	236	6	[52]
Selber JC et al.	10	68	NA	NA	[54]
Selber JC et al.	7	111	NA	NA	[56]
Fouarge A et al.	6	110	NA	5 days	[57]
**Deep inferior epigastric vein**					
Manrique OJ et al.	8 (cadaveric model)	Tapp: 56 Tep: 65	NA	NA	[69]
Fitzgerald O’Connor E et al.	265	Without CTA: 136.5 With CTA: 123.5	NA	NA	[68]

NA: Not available.

**Table 3:** Various studies delineating the safety, and efficacy of robot-assisted surgery in breast cancer patients.

Name of the Study (Robot-Assisted Breast Surgery)	Year of the Study	Objective of the Study	Total Patient Samples	References
Preliminary report of robot-assisted nipple-sparing mastectomy and immediate breast reconstruction with implant	2015	To assess suitability, safety, benefits and barriers of robot-assisted surgical procedure applied to nipple-sparing mastectomy and immediate breast reconstruction with implant.”	3 patients (Nipple-sparing mastectomy)	[83]
Robot-assisted nipple-sparing mastectomy: a feasibility study on cadaveric models	2016	To ascertain the specialized suitability of robot-assisted nipple-sparing mastectomy through lateral axillary cut utilizing corpses	Two human cadavers	[49]
Robot-assisted nipple-sparing mastectomy and immediate breast reconstruction: future perspectives for breast cancer surgery	2016	The objective of this review is to decipher the significant relevance of robotic medical procedure additionally for breast cancer patients.	Ten patients	[84]
Robotic nipple-sparing mastectomy and immediate breast reconstruction with implant: first report of surgical technique	2017	To estimate suitability, safety, benefits, and impediments of automated medical procedure to perform robot-assisted nipple-sparing mastectomy and immediate breast reconstruction with implant	3 patients (Nipple-sparing mastectomy)	[48]
Robot-assisted nipple-sparing mastectomy for the treatment of breast cancer: feasibility and safety study	2017	To ascertain the results of the initial 29 sequential robot-assisted nipple-sparing mastectomies and immediate breast reconstruction with implant methods performed and analyzed suitability, reproducibility and safety	Twenty-four patients.	[50]
Robot-assisted nipple-sparing mastectomy with immediate prosthetic breast reconstruction: a preliminary study	2017	The objective of this is to examine suitability of robot-assisted nipple-sparing mastectomy with immediate prosthetic breast reconstruction on the initial 50 consecutive cases carried out in GustaveRoussy.	50 patients (Robotic nipple-sparing mastectomy) with immediate prosthetic breast reconstruction	[50]
Robot-assisted nipple-sparing mastectomy and immediate breast reconstruction with gel implant	2018	To describe the primary experience and outcomes of robot-assisted nipple-sparing mastectomy and immediate prosthetic breast reconstruction with gel implant	Fifteen patients	[85,86]
Robot-assisted nipple-sparing mastectomy and immediate breast reconstruction with gel implant: technique, preliminary results and patient-reported cosmetic outcome	2018	To elucidate the primary experience and outcomes of robot-assisted nipple-sparing mastectomy and ‘immediate prosthetic breast reconstruction’ with gel implant	Twenty-two patients	[85]
The learning curve of robot-assisted nipple-sparing mastectomy for breast cancer: an analysis of consecutive 39 procedures with cumulative sum plot	2018	To describe the primary experience of robot-assisted nipple-sparing mastectomy in the management of breast cancer and examine the learning curve from the same surgeon	35 patients (robot-assisted nipple sparing mastectomy)	[87]
Robot da Vinci Xi robot-assisted nipple-sparing mastectomy: first clinical report	2018	To depict the surgical procedure and postoperative result of the first case of robot-assisted nipple-sparing mastectomy with da Vinci robot-assisted surgery	Forty-six year old patient	[49]
Robot-assisted nipple-sparing mastectomy with immediate prosthetic breast reconstruction: surgical technique	2018	To describe suitable robot-based breast surgery strategies, the authors have created several conclusions acquired from over 60 methodologies	Thirty-two patients	[86]
Robot-assisted prophylactic nipple-sparing mastectomy with immediate prosthetic breast reconstruction: a prospective study	2018	To describe the possibility and safety of robot-assisted nipple-sparing mastectomy with immediate prosthetic breast reconstruction	Thirty-three patients	[88]
Technique for single axillary incision robot-assisted quadrantectomy and immediate partial breast reconstruction with robot-assisted latissimusdorsi flap harvest for breast cancer: a case report	2018	To describe primary experience and clinical reports of robot-assisted quadrantectomy and immediate prosthetic breast reconstruction with robot-assisted latissimus dorsi flap harvest	Twenty-eight year old patient	[89]
Robot-assisted deep inferior epigastric artery perforator flap abdominal harvest for breast reconstruction: a case report	2018	To describe the utilization of a robot to gather the deep inferior epigastric vein in a deep inferior epigastric perforator flap-based breast reconstruction	Fifty-one year old patient	[90]
Early experiences with robot-assisted prosthetic breast reconstruction	2019	In this study, authors described a few patients with invasive ductal carcinoma who went through robot-assisted nipple-sparing mastectomy and implant-based immediate breast reconstruction with good clinical outcomes.	Four patients	[91]
Breast cancer robot-assisted nipple-sparing mastectomy: evaluation of several surgical procedures and learning curve	2019	To describe suitability of robot-assisted nipple-sparing mastectomy and evaluate the standard surgical procedure and learning curve threefold.	Twenty-seven patients	[92]

**Table 4:** The pros and cons of robot-assisted surgery compared to conventional surgery.

Type of Surgery	Pros	Cons
Conventional surgery	Economical Effective	2-dimensional Occurrence of numbness Less degree of freedom Occurrence of fulcrum effect, and a higher physiologic tremor
Robot assisted surgery	3-dimensional Exhibits less degree of freedom up to 7 degrees. No physiologic tremors. No fulcrum effect. Possibility of Telephone surgery.	No numbness Expensive More time consumption to execute the surgery. Chances of malfunctioning of robots. Highly trained personnel required to execute the procedure.

## Data Availability

Not applicable.
